# Mirtazapine is a functional antagonist at cardiac human H_1_-histamine receptors

**DOI:** 10.1007/s00210-025-04908-z

**Published:** 2025-12-20

**Authors:** Thanh Hoai Pham, Jonas M. A. Schlicht, Britt Hofmann, Uwe Kirchhefer, Joachim Neumann, Ulrich Gergs

**Affiliations:** 1 Institute for Pharmacology and Toxicology, Medical Faculty, Martin-Luther-University Halle-Wittenberg, Magdeburger Straße 4, 06097 Halle (Saale), Germany; 2 Department of Cardiac Surgery, Mid-German Heart Centre, University Hospital Halle, Ernst-Grube-Straße 40, 06097 Halle (Saale), Germany; 3 Institute for Pharmacology and Toxicology, University Hospital Münster, University Münster, Domagkstraße 12, 48149 Münster, Germany

**Keywords:** Mirtazapine, H_1_-histamine receptor, Heart, Inotropy, Chronotropy

## Abstract

Mirtazapine is an atypical tetracyclic antidepressant drug that binds to several monoamine neurotransmitter receptors. For instance, mirtazapine binds to H_1_-histamine receptors in vitro and in the brain of patients in vivo. Here, we hypothesize that mirtazapine is an antagonist at human cardiac H_1_-histamine receptors. To test this hypothesis, we measured force of contraction in isolated electrically stimulated (1 Hz) left atrial preparations (LA) and spontaneously beating right atrial preparations (RA) from adult transgenic mice with cardiomyocyte-specific overexpression of the human H_1_-histamine receptor (H_1_-TG). These findings were compared with those in wild-type littermate mice (WT). Finally, we measured the force of contraction in isolated electrically stimulated right atrial muscle strips (HAP) gotten from adult patients who were undergoing bypass surgery. Mirtazapine concentration- and time-dependently decreased H_1_-histamine receptor-stimulated force of contraction in LA and RA from H_1_-TG. Importantly, mirtazapine (starting at 100 nM) concentration- and time-dependently decreased H_1_-histamine receptor-stimulated force of contraction in HAP. We thus conclude that mirtazapine can antagonize human cardiac H_1_-histamine receptors at therapeutic drug concentrations, and thus, mirtazapine may have cardiac untoward effects in psychiatric patients.

## Introduction

Mirtazapine is an antagonist for several monoamine neurotransmitters. The peculiarity of mirtazapine is that, unlike older antidepressant drugs, it does not inhibit any transporter proteins of neurotransmitters, neither serotonin nor noradrenaline transporters as is typical for drugs like amitriptyline (Anttila and Leinonen 2001). Mirtazapine has therapeutic plasma concentrations in psychiatric patients from 151 to 301 nM (Appl et al. 2012). The therapeutic effect of mirtazapine probably results from several mechanisms (Anttila and Leinonen 2001): Mirtazapine is an antagonist at α_2_-adrenoceptors, at several serotonin receptors, and at several histamine receptors. Here, we focus only on the effect of mirtazapine as a potent antagonist at H_1_-histamine receptors in the human heart. Others reported that mirtazapine binds to H_1_-histamine receptors in vitro (K_i_-value of 8.8 molar concentration) and acts antagonistically at H_1_-histamine receptors in transfected cells (Appl et al. 2012). Others have reported that mirtazapine binds to H_1_-histamine receptors in the brain of living humans using radioactive tracers and positron emission tomography (Enomoto et al. 2012, Sato et al. 2013, Hassanein et al. 2024). Of all the receptors to which mirtazapine binds, mirtazapine has the highest affinity at H_1_-histamine receptors (Anttila and Leinonen 2001; Bakker et al. 2007; Appl et al. 2012). However, it is unknown whether mirtazapine might be an antagonist at human cardiac H_1_-histamine receptors.

**Fig. 1:**
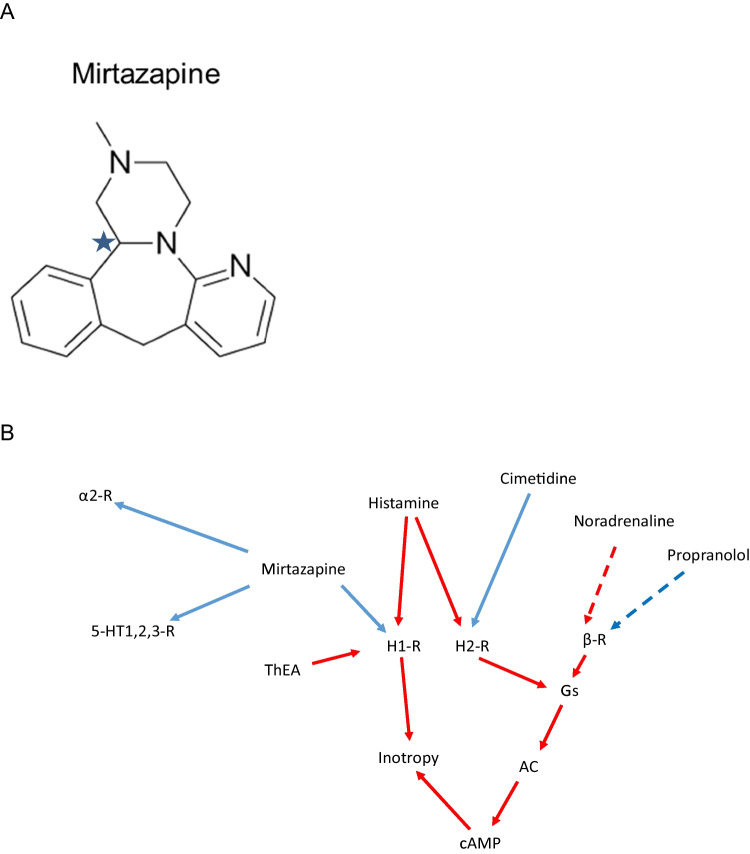
**A** Structural formula of mirtazapine. It contains a chiral carbon atom, here indicated by an arrow. Clinically, the racemate is used, and there we also used throughout the present study racemic mirtazapine. **B** Hypothetical action of mirtazapine. Mirtazapine might block H_1_-histamine receptors (H_1_-R) stimulated by histamine and 2-(2-thiazolyl)-ethylamine (ThEA) in the sarcolemma of cardiomyocytes. If mirtazapine released noradrenaline, this would stimulate β-adrenoceptors (β-AR, inhibited by propranolol) in cardiomyocytes. Histamine can also stimulate cardiac H_2_-histamine receptors (H_2_-R, blocked by cimetidine). Adenylyl cyclases (AC) may increase intracellular cAMP. H_1_-histamine receptor (H_1_-R) may increase the force of contraction. Mirtazapine can inhibit brain α_2_-adrenoceptors and several brain serotonin receptors (5-HT1,2,3-R) such as 5-HT_1_-receptors, 5-HT_2_-receptors, and 5-HT_3_-receptors

Mirtazapine, a piperazinoazepine, has four cyclic rings in its structural formula and has a chiral carbon atom (Fig. 1A). Despite its chirality, mirtazapine is used as a racemic drug, and therefore, we studied in this report racemic mirtazapine. Mirtazapine was developed starting in 1989 and was approved as an anti-depressive drug in the USA and Europe in the 1990s. The anti-depressant effects of mirtazapine may be due to an inhibitory action on α_2_-adrenoceptors in the brain. These inhibitory effects of mirtazapine at central α_2_-adrenoceptors lead to high serotonin levels in synaptic cleft. This may be advantageous if it is assumed that depression is due to a low level of serotonin at central serotonin receptors (Anttila and Leinonen 2001). In addition, mirtazapine also binds to most serotonin receptors with highest affinity (Ki-values in brackets in negative decadic logarithmic molar concentrations), to 5-HT_2A_- (8.2), 5-HT_2C_- (7.9), and 5-HT_3_-(8.1)-serotonin receptors. This serotonin receptor binding profile might explain the antiemetic effect (blockage of 5-HT_3_-receptors) and the weight gain (blockage of 5-HT_2C_-receptors). Mirtazapine therefore binds preferentially to the H_1_-histamine receptors at therapeutic plasma concentrations before other receptors such as serotonin receptors are occupied (Fig. 1B). It is assumed that the blockage of H_1_-histamine receptor antagonists in the brain explains the somnogenic effects and weight-increasing effects of mirtazapine (Davis and Wilde 1996, Behlke et al. 2020). Hence, antagonism at H_1_-histamine receptor of mirtazapine occurs in the living human body (Behlke et al. 2020). It can be assumed that this also occurs in the heart (Fig. 1B).

Histamine acts via H_1_-, H_2_-, H_3_-, and H_4_-histamine receptors (Panula et al. 2015, Neumann et al. 2021). Only H_1-_ and H_2_-histamine receptors are detectable and functional on cardiomyocytes (Neumann et al. 2021). Mirtazapine does not bind to β-adrenoceptors (Proudman et al. 2022). Hence, any negative inotropic effect of mirtazapine cannot be explained by blockage of β-adrenoceptors. To better understand this process, we have recently generated and characterized a transgenic mouse model with cardiospecific overexpression of the H_1_-histamine receptor (H_1_-TG). In pilot experiments, we used H_1_-TG to study in principle whether mirtazapine can antagonize H_1_-histamine receptors. We had previously shown that ThEA exerts a positive inotropic effect via H_1_-histamine receptors under appropriate conditions, which we also used here (Pham et al. 2024). Under these well-established conditions, we asked here whether or not mirtazapine might exert a negative inotropic effect at clinically relevant concentrations. To the best of our knowledge, inotropic effects of mirtazapine on H_1_-histamine receptors in the human heart are currently speculative. In order to translate our findings from transgenic mice on mirtazapine potentially to the clinic, we also decided to investigate the effects of mirtazapine on the force of contraction in the human heart. For this purpose, we employed right atrial strips which we had gotten from our cardiac surgery operating room.

We investigated the hypotheses: firstly, mirtazapine blocks H_1_-histamine receptor-stimulated contractility in atrial preparations from H_1_-TG and WT. Secondly, mirtazapine blocks H_1_-histamine receptor-stimulated contractility in the isolated human atrium via H_1_-histamine receptors.

## Materials and methods

### Transgenic mice

Animals were kept and bred in compliance with permit of the animal local welfare committee (Veterinäramt der Stadt Halle, permission # I8M9). The generation and initial characterization of the transgenic mice (H_1_-TG) have been described before (Rayo et al. 2024). The mice were generated by pronuclear DNA injection of the human H_1_-receptor cDNA which had been inserted into a mouse cardiac α-myosin heavy chain promoter expression cassette. This leads to specific cardiac overexpression of the transgene, as we have shown with several methods (Rayo et al. 2024). For the reported data, adult (mean value about 160 days) H_1_-histamine receptor transgenic mice and WT littermates of both sexes were utilized.

### Contractile studies in mice

Housing, handling, raising, and sacrificing of mice complied with local regulations (permission # I8M9). Mice were sacrificed by cervical dislocation. Then, the thorax was cut open. The heart as a whole was dissected from surrounding tissue and transferred to a glass Petri dish filled with the following modified Tyrode’s solution, which was held at ambient temperature. In this modified Tyrode’s solution, the right (RA) or left atrial preparations (LA) were cut with single scissor strokes from the heart. Then, LA and RA were moved to double-barrelled organ baths (containing 10 ml buffer volume) as repeatedly reported (e.g., Gergs et al. 2017). The modified Tyrode’s solution contained in millimolar concentrations (mM): 119.8 NaCI, 5.4 KCI, 1.8 CaCl_2_, 1.05 MgCl_2_, 0.42 NaH_2_PO_4_, 22.6 NaHCO_3_, 0.05 Na_2_EDTA, 0.28 ascorbic acid, and 5.05 glucose. Ascorbic acid is used here as an antioxidant to maintain the activity of, for instance, isoprenaline or histamine. The solution was continuously gassed with 95% O_2_ and 5% CO_2_ to maintain the pH 7.4 in the organ baths. Spontaneously beating RA, we used to monitor chronotropic and inotropic effects. LA were studied to measure force under isometric conditions at pre-defined constant beating rate and thus to obviate any Treppe phenomenon. LA were lengthened to an optimal length that allowed maximal generation of force of contraction (3–4 milli Newton). LA were activated (one Hertz) with electrical impulses through platinum electrodes. These were rectangular impulses (5 ms of duration) of direct currents that came from a Grass stimulator SD 9 (Quincy, Massachusetts, USA). Voltage for stimulation of LA lies between 5 and 10 V, about 10% above the voltage to start contractions. The alterations of the force using the transducer (Hellige, Freiburg, Germany) were connected to the input for a bridge amplifier (AD Instruments) and digitized and stored on a commercial personal computer (Dell, Halle, Germany). The signals were quantified (to assess force in milli Newton, its first derivate, the time to peak tension, and the time of relaxation) using a commercial software (Lab Chart 8, ADInstruments, Oxford, England).

### Contractile studies on human preparations

In the contractile measurements in HAP, we used the same organ bath set-up and modified Tyrode’s solution as mentioned above in the mouse experiments. The procedures followed our published work (e.g., Gergs et al. 2009, 2017). Thus, HAP was moved from cardiac surgery into our laboratory, sitting in modified Tyrode’s solution within 30 min. Using a dissecting microscope (Hund, Wetzlar, Germany), samples were cut into small trabecular muscle pieces. These muscle strips were pierced with metal hooks at each end of the muscle and transferred to the glass organ baths where they were mounted vertically on a plastic rod. Like LA (vide supra), also human muscle strips were electrically paced at 1 Hz using rectangular impulses of 5 ms duration and 10% above the voltage necessary for initiation of the beating (here around 10 V). Human muscle strips were lengthened to the maximum of the force-distension relationship. Signals were amplified and quantified as described above for mice atria. The HAP were secured from male patients of 58–69 years of age. All the patients endured severe coronary disease (two and three vessel diseases). The cardiac drug therapy encompassed acetylsalicylic acid or other antithrombotic agents, apixaban or other anticoagulants, furosemide or other diuretic drugs, and metoprolol or other β-adrenoceptor antagonists. Additional drugs in these patients were (in alphabetical order) amlodipine, atorvastatin, budesonide, candesartan, clonidine, dapaglifloxin, empaglifoxin, eplerenone, ezetimibe, insulin, lamotrigine, metformin, pantoprazole, perindopril, pregabalin, ramipril, thiamazole, sacubitril, simvastatin, sitagliptin, spironolactone, and valsartan. Cardiac comorbidities included in addition to angina pectoris also hypertension, aortic stenosis, and atrial fibrillation. Additional morbidities that our patients presented (in alphabetical order) were alcoholism, bladder cancer, epilepsia, gastritis, hyperlipidemia, hyperthyroidism, hyperuricemia, obesity, prostatomegaly, prostate cancer, renal insufficiency, shingles, testicular cancer, and type 2 diabetes. In some experiments, we initially or finally applied receptor antagonists to the organ baths and then the β-adrenoceptor agonist isoprenaline as a positive control, as delineated in the figure legends. As described in the appropriate legends, in mouse atria and HAP, mirtazapine was applied cumulatively. This occurred without or after pre-incubation with 0.4 µM propranolol, 100 µM cimetidine, 10 µM ThEA, or 100 µM histamine. These experiments complied with the local ethics commission (permission # hm-bü). All patients gave written informed content for this study.

### Data analysis

Data shown are means ± SEM. Statistical significance was evaluated by analysis of variance followed by Bonferroni’s *t*-test or Student’s *t*-test using GraphPad Prism® 9. A *p*-value < 0.05 was considered as significant. Normality was also tested and confirmed using GraphPad Prism® 9.

### Drugs and materials

Histamine, cimetidine, mepyramine, racemic mirtazapine, and propranolol were from Merck, Dreieich, Germany. 2-(2-Thiazolyl)-ethylamine (ThEA) was purchased from BLD Pharmatech GmbH, Reinbeck, Germany. All other chemicals were of analytical grade. Demineralized water was used throughout the experiments. Stock solutions were prepared daily.

## Results

### Contraction in left atrium of H_1_-TG

**Fig. 2:**
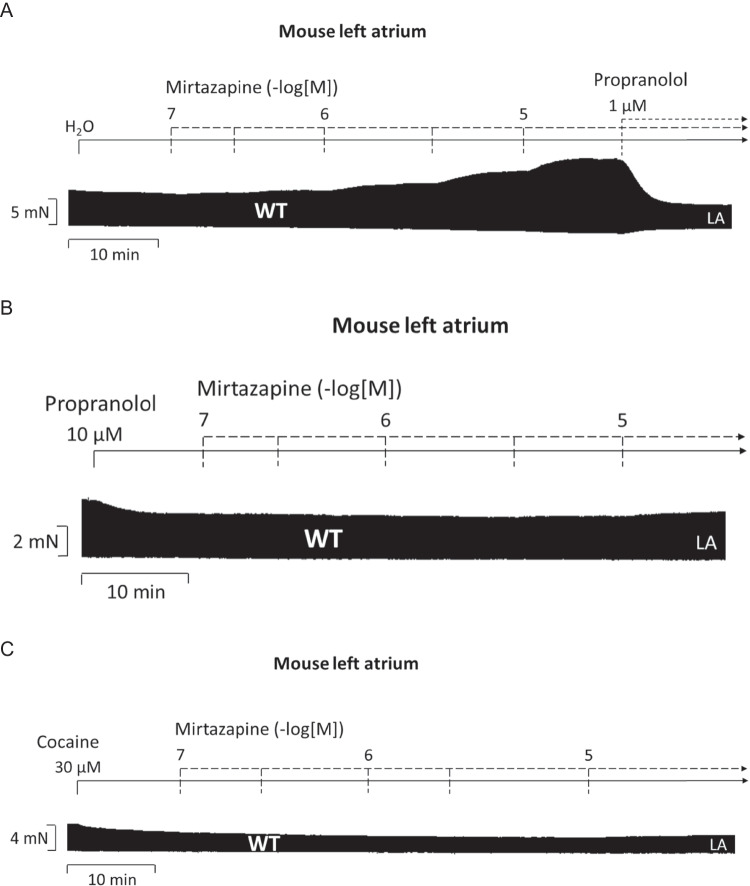

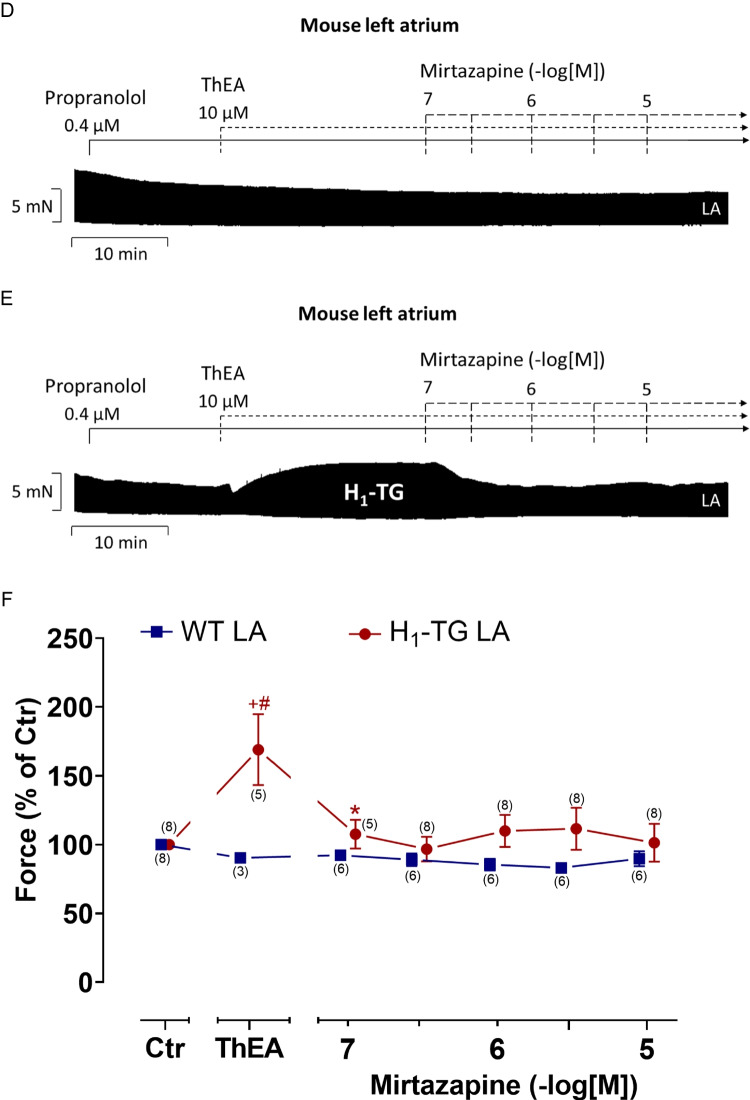
Mirtazapine antagonizes the positive inotropic effect of ThEA in left atrial preparations of H_1_-TG mice. Original recordings depicting the effect of mirtazapine alone on force of contraction and subsequently applied propranolol in order to block β-adrenoceptors (**A**), with firstly applied propranolol (**B**) and firstly applied cocaine (**C**) in isolated electrically stimulated left atrial preparations (LA) from wild-type (WT) mice. Original recordings of the effect of mirtazapine after propranolol and ThEA application in LA from WT are seen in **D** and from mice with cardiac overexpression of the human H_1_-histamine receptor (H_1_-TG) in **E**. Horizontal bars indicate time axis in minutes (min) and vertical bars in milli Newton (mN). In addition, the concentration-dependent effects of mirtazapine on force of contraction in LA after propranolol (Ctr) and ThEA addition were summarized for H_1_-TG and their WT littermates in percentage (WT 100% ≙ 3.73 mN, H_1_-TG 100% ≙ 2.13 mN; **F**) Abscissae indicate concentrations of mirtazapine in negative decadic logarithmic molar concentrations. Ctr indicates pre-drug values. ThEA indicates the effect of 10 µM ThEA. The number in brackets indicates the number of experiments. * indicates the first significant (*p* < 0.05) difference vs. ThEA (ANOVA Bonferroni), ^+^ indicates the significant (*p* < 0.05) difference vs. Ctr (Student’s *t*-test), and ^#^ indicates the significant (*p* < 0.05) difference vs. WT (Student’s *t*-test). Some error bars do not appear because they are shorter than the size of the symbols

**Fig. 3:**
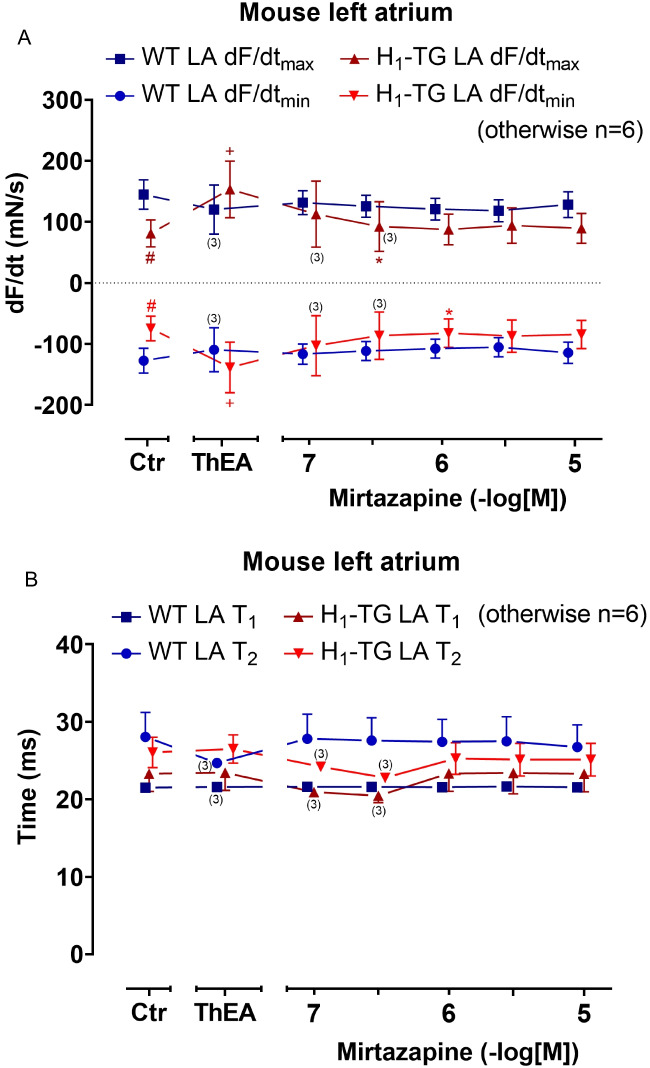
Mirtazapine shortens the rate of tension development and relaxation in the left atrium in H_1_-TG after ThEA addition. Concentration–response curves for mirtazapine after ThEA addition in electrically stimulated mouse LA from H_1_-TG and WT mice (**A**). We added 0.4 µM propranolol to the organ bath in order to block β-adrenoceptors. ThEA induced an increase in the rate of tension development and relaxation in H_1_-TG mice, which could be antagonized by mirtazapine (**A**). **B** Time to peak tension (T_1_) and time of relaxation (T_2_) curve of H_1_-TG and WT mice. Ordinate in **A** depicts the rate of tension development (dF/dt_max_) and the rate of tension relaxation (dF/dt_min_) in milli Newton per second (mN/s), whereas ordinate in **B** depicts time to peak tension (T_1_) and time of relaxation (T_2_) in milliseconds (ms). Abscissae indicate concentrations of mirtazapine in negative decadic logarithmic molar concentrations. Ctr indicates pre-drug values. ThEA indicates the effect of 10 µM ThEA. Numbers in brackets indicate the number of experiments. * indicates the first significant (*p* < 0.05) difference vs. ThEA (ANOVA Bonferroni), ^+^ indicates the significant (*p* < 0.05) difference vs. Ctr (Student’s *t*-test), and ^#^ indicates the significant (*p* < 0.05) difference vs. WT (Student’s *t*-test). Some error bars do not appear because they are shorter than the size of the symbols

We cumulatively gave mirtazapine (0.1 to 10 µM) in left atrial preparations from wild-type mice (WT, Fig. 2A). Mirtazapine exhibited a time- and concentration-dependent positive inotropic effect that was antagonized by subsequently applied 1 µM propranolol, which completely reversed the positive inotropic effect of mirtazapine (Fig. 2A). As a control, in a separate study, we firstly applied 10 µM propranolol and added thereafter increasing concentrations of mirtazapine (Fig. 2B). Then, no positive inotropic effect of mirtazapine was observed in WT. So far, these data suggested the possibility that mirtazapine is a direct or indirect sympathomimetic agent. In order to decide between these possibilities, in a separate atrium, we first applied 10 µM cocaine in order to inhibit the uptake of noradrenaline (Fig. 2C). Again, no positive inotropic effect of mirtazapine was observed. As expected and published by us before (Pham et al. 2024), ThEA alone had no positive inotropic effect in LA from WT (Fig. 2D). We have explained this lack of inotropic effect with a very low protein expression of the H_1_-histamine receptor or with ineffective coupling to intracellular messengers (Pham et al. 2024, 2025a). In contrast, in left atrial preparations from H_1_-TG, a positive inotropic effect after ThEA application could be observed. Such a positive inotropic effect of ThEA was reported before in LA from H_1_-TG (Pham et al. 2024, 2025b). The main new finding here is that we could antagonize this positive inotropic effect of ThEA by cumulatively applying mirtazapine (Fig. 2E). Please note in the original recording that already 100 nM mirtazapine induced a negative inclination in the recording (Fig. 2A). We started here with 100 nM mirtazapine because this is just below the therapeutic drug concentration of mirtazapine in clinical studies, and hence, this concentration is clinically relevant. Please also note that in these studies, we always included propranolol (e.g., Fig. 2E). These data are summarized with regard to the force of contraction for H_1_-TG and WT in Fig. 2F in percentage. In LA from H_1_-TG but not from WT, ThEA increased the force of contraction. Furthermore, mirtazapine starting at 100 nM not only reduced the force of contraction (Fig. 2F) but also reduced the absolute values of the maximum rate of tension development and the minimum rate of relaxation after ThEA application in H_1_-TG (Fig. 3A), whereas no influence in time to peak tension and time of relaxation could be observed (Fig. 3B). The absence of an effect on time to peak force and time of relaxation of ThEA per se is identical with our previously published data in these mice (Pham et al. 2024, Pharma et al. 2025a, Pham et al. 2025b): it is typical of H_1_-histamine-receptor mediated effects. In contrast, H_2_-mediated receptor mediated effects are accompanied by a reduced time to relaxation because they involve the cAMP pathway (Fig. 1B, Gergs et al. 2019, Pham et al. 2025b).

### Contraction in right atrium of H_1_-TG

**Fig. 4:**
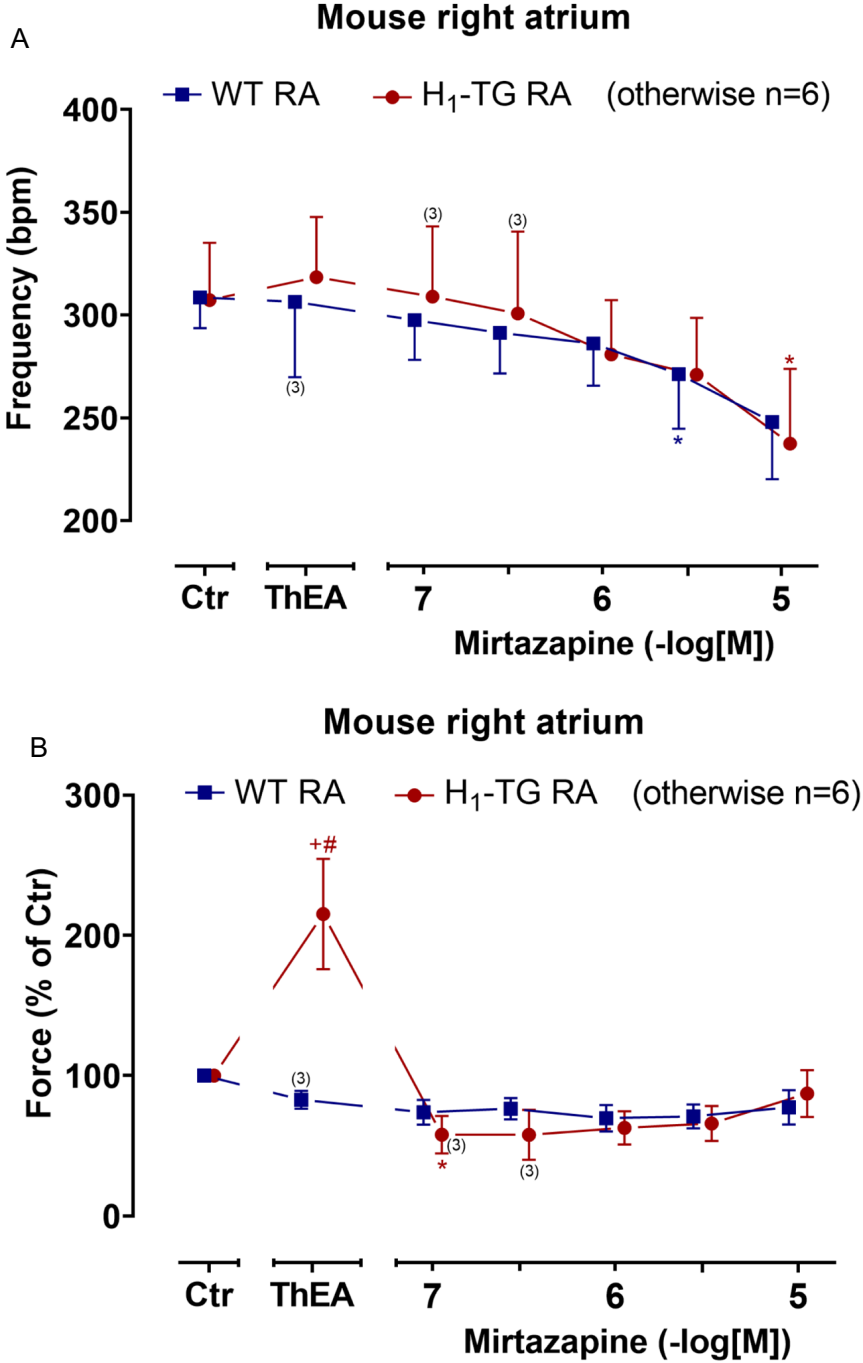
Mirtazapine decreases the beating rate and force of contraction in the right atrium of H_1_-TG and WT mice after ThEA addition. Summarized concentration response curves for the effect of mirtazapine on the beating rate (in beats per minute, bpm) after ThEA addition in spontaneously beating isolated right atrial (RA) preparations from H_1_-TG and their WT littermates (**A**) and on force of contraction in % (WT 100% ≙ 1.33 mN, H_1_-TG 100% ≙ 0.54 mN; **B**). We also added 0.4 µM propranolol to the organ bath in order to block β-adrenoceptors. Ordinate in **A** depicts beats per minute (bpm). Abscissae indicate concentrations of mirtazapine in negative decadic logarithmic molar concentrations. Ctr indicates pre-drug values. ThEA indicates the effect of 10 µM ThEA. The number in brackets indicates the number of experiments. * indicates the first significant (*p* < 0.05) difference vs. ThEA (ANOVA Bonferroni), ^+^ indicates the significant (*p* < 0.05) difference vs. Ctr (Student’s *t*-test), and ^#^ indicates the significant (*p* < 0.05) difference vs. WT (Student’s *t*-test). Some error bars do not appear because they are shorter than the size of the symbols

**Fig. 5:**
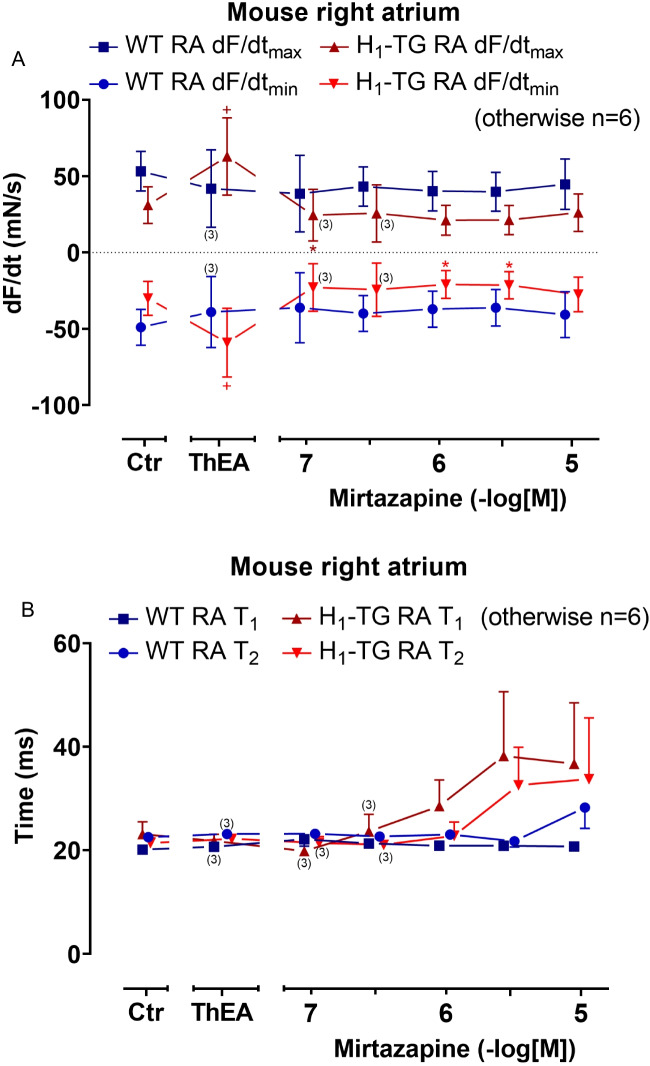
Mirtazapine shortens the rate of tension development and relaxation and increases time to peak tension and time of relaxation in the right atrium in H_1_-TG after ThEA addition. Concentration response curves for mirtazapine after ThEA addition in spontaneously beating right atrium (RA) from H_1_-TG and WT mice. We added 0.4 µM propranolol to the organ bath in order to block β-adrenoceptors. **A** Rate of tension development (dF/dt_max_) and rate of relaxation (dF/dt_min_) from H_1_-TG and WT mice. **B** Time to peak tension (T_1_) and time of relaxation (T_2_) curve of H_1_-TG and WT mice. Ordinate in **A** depicts the rate of tension development (dF/dt_max_) and the rate of tension relaxation (dF/dt_min_) in milli Newton per second (mN/s), whereas ordinate in **B** depicts time to peak tension (T_1_) and time of relaxation (T_2_) in milliseconds (ms). Abscissae indicate concentrations of mirtazapine in negative decadic logarithmic molar concentrations. Ctr indicates pre-drug values. ThEA indicates the effect of 10 µM ThEA. The number in brackets indicates the number of experiments. * indicates the significant (*p* < 0.05) difference vs. ThEA (ANOVA Bonferroni), and ^+^ indicates the significant (*p* < 0.05) difference vs. Ctr (Student’s *t*-test). Some error bars do not appear because they are shorter than the size of the symbols

Then, we studied the right atrial function. Mirtazapine time- and concentration-dependently decreased the beating rate in WT and H_1_-TG mice (Fig. 4A). In the same preparations, we also measured the force of contraction and noted results qualitatively similar to those in LA (Fig. 4B). ThEA only induced a positive inotropic effect in right atrial preparations from H_1_-TG but not from WT, which was reduced by cumulatively applied mirtazapine (Fig. 4B). Furthermore, ThEA also increased the rate of tension development and the rate of relaxation in RA from H_1_-TG only, which could be reversed by cumulatively applied mirtazapine (Fig. 5A). ThEA did not affect time to peak tension and time of relaxation in H_1_-TG and WT, but interestingly, mirtazapine raised time to peak tension in H_1_-TG and time of relaxation in H_1_-TG and WT (Fig. 5B).

### Contractions in human right atrial preparations

**Fig. 6:**
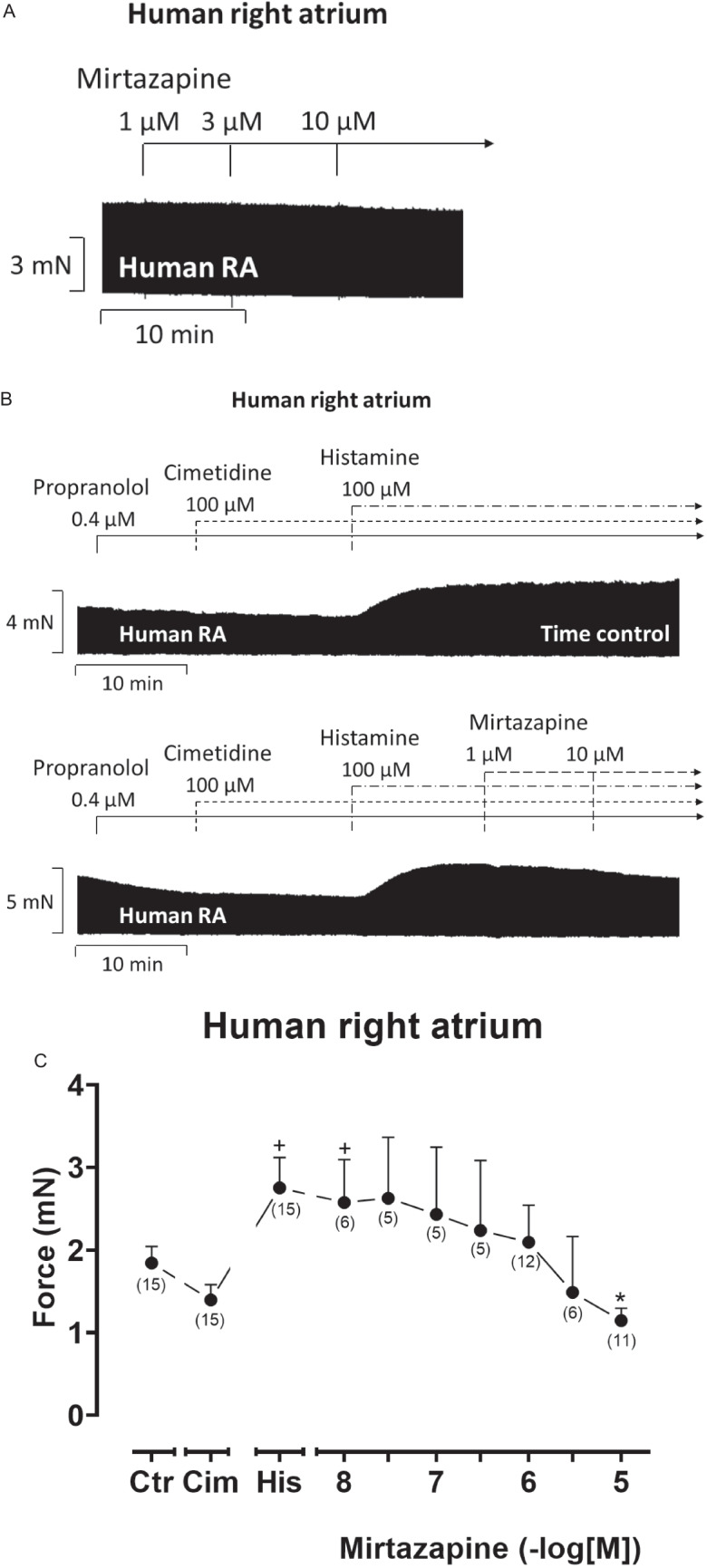
Mirtazapine antagonizes the positive inotropic effect of histamine in human right atrial preparations. Original recordings depicting the effect on force of contraction of mirtazapine alone (**A**), of histamine alone as time control (**B**, top), and of cumulatively applied mirtazapine after histamine addition (**B**, below) in electrically stimulated (1 Hz) human atrial preparations (HAP). Horizontal bars indicate the time axis in minutes (min) and vertical bars in milli Newton (mN). In addition, the concentration-dependent effect of cumulatively applied mirtazapine after histamine addition on force of contraction was summarized in absolute values in mN (**C**). In samples, we added 0.4 µM propranolol to the organ bath in order to block β-adrenoceptors as well as 100 µM cimetidine in order to block histamine H_2_-receptors. Ordinate in **C** depicts the developed force of contraction in milli Newton (mN). Abscissae indicate concentrations of mirtazapine in negative decadic molar concentrations. Ctr indicates pre-drug values. Cim indicates the effects of 100 µM cimetidine. His indicates the effects of 100 µM histamine. The number in brackets indicates the number of experiments. * indicates the significant (*p* < 0.05) difference vs. His (ANOVA Bonferroni), and ^+^ indicates the significant (*p* < 0.05) difference vs. Cim (Student’s *t*-test). Some error bars do not appear because they are shorter than the size of the symbols

**Fig. 7:**
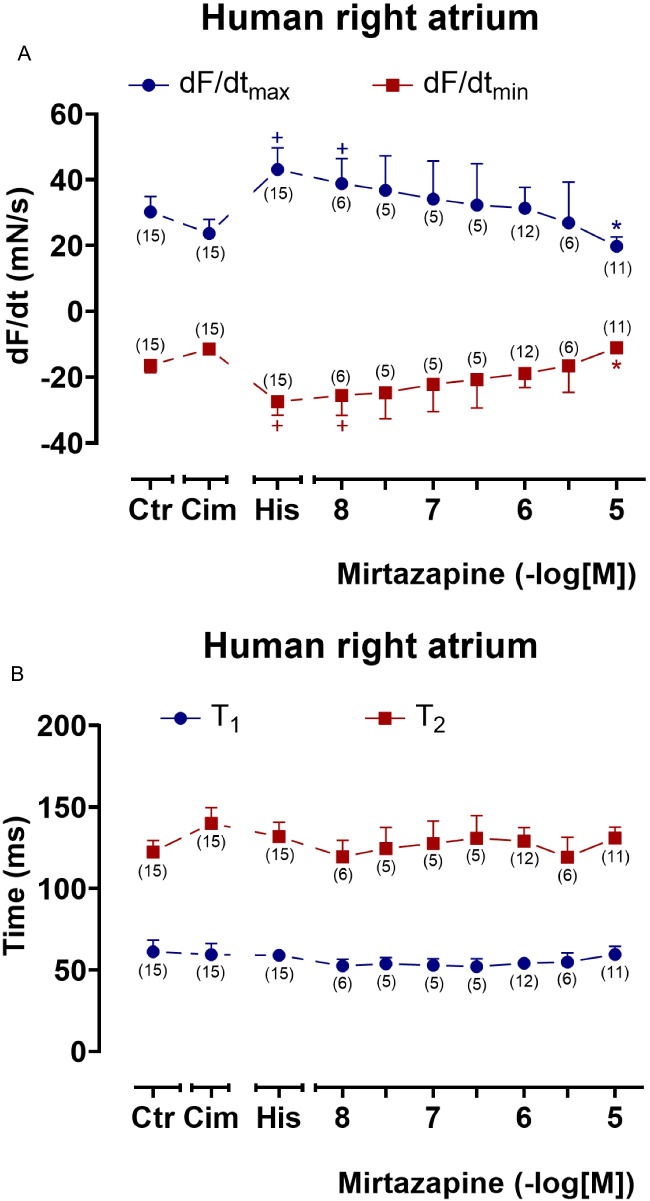
Mirtazapine shortens the rate of tension development and relaxation in HAP after histamine addition. Concentration response curves for cumulatively applied mirtazapine after histamine addition in electrically stimulated (1 Hz) HAP. We added 0.4 µM propranolol to the organ bath in order to block β-adrenoceptors as well as 100 µM cimetidine in order to block histamine H_2_-receptors. **A** Rate of contraction (dF/dt_max_) and rate of relaxation (dF/dt_min_). **B** Time to peak tension (T_1_) and time of relaxation (T_2_) curve. The highest concentration of mirtazapine antagonized the rate of contraction and the rate of relaxation. Ordinate in **A** depicts the rate of tension development (dF/dt_max_) and the rate of tension relaxation (dF/dt_min_) in milli Newton per second (mN/s), ordinate in **B** depicts the time to peak tension (T_1_) and the time of relaxation (T_2_) in milliseconds (ms). Abscissae indicate concentrations of mirtazapine in negative decadic molar concentrations. Ctr indicates pre-drug values. Cim indicates the effects of 100 µM cimetidine. His indicates the effects of 100 µM histamine. The number in brackets indicates the number of experiments. * indicates the first significant (*p* < 0.05) difference vs. His (ANOVA Bonferroni), ^+^ indicates the significant (*p* < 0.05) difference vs. Cim (Student’s *t*-test). Some error bars do not appear because they are shorter than the size of the symbols

In isolated human right atrial preparations (HAP), we first investigated the effect of mirtazapine alone. In contrast to LA in mice, 1, 3, and 10 µM mirtazapine showed no positive inotropic effect in HAP (original recording in Fig. 6A). This clearly shows that there are species differences in the inotropic effects of mirtazapine and that, therefore, studies in mice cannot replace studies in human cardiac tissue. When HAP were beating in the presence of propranolol and 100 µM cimetidine (to block endogenous H_2_-histamine receptors in HAP which would be otherwise stimulated, Pham et al. 2025a), additionally applied 100 µM histamine had a positive inotropic effect (Fig. 6B, top). In a previous publication, we had shown that the positive inotropic effect to 100 µM histamine under these conditions was H_1_-histamine receptor-mediated, as it could be antagonized by mepyramine, a H_1_-histamine receptor antagonist (Pham et al. 2024, 2025b). Based on this established protocol for the positive inotropic effect via H_1_-histamine receptors in HAP, we then added mirtazapine. Hereafter, we noted a concentration- and time-dependent negative inotropic effect of mirtazapine (Fig. 6B, below). When one compares this effect of mirtazapine with a time control, it became obvious that this effect was not the result of a run-down of the HAP (Fig. 6B, top tracing). The negative inotropic effect of mirtazapine was apparent in absolute force (Fig. 6C). The effect was mirrored with respect to the first derivative of force with respect to time (Fig. 7A). In contrast to H_2_-histamine receptor stimulation, the stimulation of the H_1_-histamine receptor in mammalian hearts including human hearts is not accompanied by a shortening of T_1_ and T_2_ (Pham et al. 2025a). This was also observed in the present set of experiments (Fig. 7B). Additionally applied mirtazapine failed to affect these parameters (Fig. 7B).

## Discussion

The new finding of this study lies in the observation that mirtazapine antagonized the atrial positive inotropic effect of H_1_-histamine receptors. We come to this conclusion for the following reasons: first, we used an established animal model (H_1_-TG) to study the positive inotropic effect of histamine via H_1_-histamine receptors (described in Rayo et al. 2024). Under conditions that we described before in detail (Rayo et al. 2024, Pham et al. 2024), we detected a H_1_-histamine receptor-mediated a positive inotropic effect which was antagonized by mirtazapine. These data convincingly show that mirtazapine antagonized the effects of histamine on force of contraction via H_1_-histamine receptor in the mammalian heart. This finding supports our view that this also applies to the human atrium. Here, we also used previously delineated conditions to study H_1_-histamine receptor stimulation in HAP (Pham et al. 2024). The difference that is of interest here is that in H_1_-TG, there is no positive inotropic effect of histamine mediated via H_2_-receptor. These effects are also missing in WT. This is because H_1_-TG can be regarded as WT mice with respect to the H_2_-histamine receptor. Two lines of evidence support this interpretation of our data. Firstly, when we generated transgenic mice that harbor the human H_2_-histamine receptor in the heart, our methods can detect a positive inotropic effect of histamine that is sensitive to cimetidine, an H_2_-histamine receptor antagonist. Secondly, others and we have shown that histamine itself also exerts a positive inotropic effect in HAP, which is blocked by cimetidine, and on this evidence, it is H_2_-histamine receptor mediated (Ginsburg et al. 1980; Gergs et al. 2020). In other words, HAP contain both H_1_- and H_2_-histamine receptors, whereas H_1_-TG only contains H_1_-histamine receptors in their atria. Recently, we crossbred H_1_-TG and H_2_-TG to obtain H_1_xH_2_-TG. In this mouse model, histamine exerted a positive inotropic effect via both H_1_- and H_2_-histamine receptors like in the human heart (Pham et al. 2025b). Therefore, we did not need cimetidine in our studies with atria from H_1_-TG, but we did need cimetidine to suppress the positive inotropic effect via H_2_-histamine receptors in HAP. We have also previously reported that mepyramine blocks the positive inotropic effect of histamine in H_1_-TG and that mepyramine blocks the effect of histamine on force of contraction in HAP mediated by H_1_-histamine receptor stimulation (Rayo et al. 2024, Pharm et al. 2024). We now know that mirtazapine binds as an antagonist to recombinant H_1_-histamine receptors in cell culture with a similar affinity as mepyramine (Appl et al. 2012). While it is evident that histamine stimulates H_1_- and H_2_-histamine receptors, questions can be raised about the specificity of ThEA. While some consider ThEA to be H_1_-histamine receptor selective, this is not the case in the human heart: we noticed that ThEA exerts a positive inotropic effect that is both H_1_- and H_2_-histamine receptor-mediated. We concluded this from our observation that the positive inotropic effect of ThEA in HAP is partly blocked by cimetidine and partly blocked by mepyramine (Pham et al. 2024). Taken together, these findings suggest that mirtazapine blocks the positive inotropic effect in the human atrium via H_1_-receptor stimulation. Of note, there were species differences in the response to mirtazapine: in mouse left atrial preparations from WT, mirtazapine exerted a positive inotropic effect. We suggest here that this effect results from a release of noradrenaline. We conclude this from the observation that the positive inotropic effect was reversed by propranolol (Fig. 2A) and was prevented by propranolol (Fig. 2B). The positive inotropic effect in LA from WT was also prevented by cocaine (Fig. 2C). These data combined suggest that mirtazapine released noradrenaline from stores in the mouse left atrium. Somewhat unexpectedly this did not hold true in human atrial preparations. Here, mirtazapine failed to increase force of contraction on its own in HAP (Fig. 6). One explanation could be that for any drug, it is easier to release noradrenaline in mouse atrium than in human atrium. We deem this conclusion hardly convincing. In contrast, when we studied amphetamine, we noted the opposite: amphetamine failed to increase force of contraction in mouse atrium but increase force of contraction in HAP. This positive inotropic effect was blocked by propranolol and cocaine (Neumann et al. 2023a). Hence, we suggest that this effect is drug specific: mirtazapine does not release noradrenaline in HAP which makes the interpretations of the contraction experiments in HAP more clear-cut. As can be remembered from Fig. 1B, mirtazapine does not only inhibit H_1_-histamine receptors but also block α_2_-adrenoceptors and several serotonin receptors. These receptors are also biochemically detected in the heart, of note in the human atrium. However, a blocking of these receptors can hardly explain a negative inotropic effect: the only serotonin receptor namely 5-HT_4_-serotonin receptor active in human hearts only exerts a positive inotropic effect (Gergs et al. 2010; Sanders et al. 1996). The α_2_-adrenoceptor inhibits release of noradrenaline. If it is inhibited in the heart, one might lead to a positive inotropic effect: this does not explain negative inotropic effects of mirtazapine in mouse or HAP. Moreover, we noted a positive inotropic effect to clonidine, a known α_2_-adrenoceptor agonist but only via H_2_-histamine receptors (Neumann et al. 2023b). Moreover, mirtazapine was inactive in WT to reduce force of contraction suggesting that H_1_-histamine receptor stimulation is required to detect any effect of mirtazapine. These observations combined argue for a negative inotropic effect of mirtazapine via H_1_-histamine receptors in HAP.

### Clinical relevance

Mirtazapine is mainly metabolized by oxidation. This oxidation is catalyzed mainly by cytochrome CYP2D6 (Anttila and Leinonen 2001). There are patients with low activity of this enzyme (Taylor et al. 2020). There are also other drugs that inhibit the activity of this enzyme (Rüdesheim et al. 2025). Therefore, there will be patients in whom higher plasma levels than intended (beyond 300 nM: Appl et al. 2012) are to be expected. In such patients, H_1_-histamine receptors in the heart may be fully occupied by mirtazapine (the pKi value being 8.8 in negative logarithmic molar concentration: Appl et al. 2012). While mirtazapine is considered well-tolerated in clinical case studies (Davis and Wilde 1996, Anttila and Leinonen 2001, Behlke et al. 2020), these studies might be overly optimistic in light of the present findings and may be overly designed to detect minor and not frequently occurring severe side effects of mirtazapine. Furthermore, in patients with existing diseases, the side effects noted with mirtazapine could be attributed to these. Specifically, mirtazapine at therapeutic doses might affect H_1_-histamine receptors not only in the brain, which is undisputed, but also in the heart. We found a case report in which mirtazapine led to first-degree atrioventricular block and tachycardia (Cheung et al. 2020). Cardiac arrhythmias with mirtazapine are therefore not uncommon and may be worth further study.

### Limitations

We did not have access to human ventricular samples. Therefore, we cannot conclude that mirtazapine acts on the human ventricle. However, in our view, the role of H_1_-histamine receptors in the human ventricle is controversial and in itself requires further research (Neumann et al. 2023a, b): a negative inotropic effect, no effect, and a positive inotropic effect of H_1_-histamine receptor activation have been reported in isolated electrically driven muscle strips (Neumann et al. 2023a, b). Nevertheless, it is a disadvantage of the present work that we did not study the ventricular function in the human heart or in the ventricles of WT compared to H_1_-TG. Of course, we admit that for cardiac output, the left ventricle is more important than the right atrium. Hence, in the future, a study of mirtazapine in ventricular human preparations seems warranted. In addition, it merits further study to find out whether transient or persistent atrial fibrillation can alter the expression of H_1_-histamine receptors in the human atrium or human ventricle.

In conclusion, mirtazapine is an antagonist at human H_1_-histamine receptors in the isolated atrium of transgenic mice (H_1_-TG). Mirtazapine acts as an antagonist at H_1_-histamine receptors in the isolated human atrium.

## Data Availability

The data of this study are available from the corresponding author upon reasonable request.
